# Concurrent use and association of patient-reported experience and outcome measures in psychiatric and substance use disorder care: a scoping review

**DOI:** 10.3389/frhs.2025.1620809

**Published:** 2025-06-30

**Authors:** Marte Karoline Råberg Kjøllesdal, Hilde Hestad Iversen, Lina Harvold Ellingsen-Dalskau

**Affiliations:** ^1^ Department of Public Health Science, Norwegian University of Life Sciences, Ås, Norway; ^2^ Center for Evidence-Based Public Health: A Joanna Briggs Institute Affiliated Group, Ås, Norway; ^3^ Division of Health Services Research, Norwegian Institute of Public Health, Oslo, Norway

**Keywords:** mental health care, substance use disorder treatment, patient experiences, patient reported outcome measures, instruments, health care quality

## Abstract

**Background:**

Patient reported experience measures (PREMs) provide patients` perspectives on health care services received, while generic Patient reported outcome measures (PROMs) reflect their subjective well-being or quality of life. The relationship between these measures is not well understood.

**Aims:**

To assess concurrent use and relationship of PREMs and PROMs In psychiatric and substance use disorder care, to inform how they best can be used concurrently in measuring quality of care from the patient perspective.

**Methods:**

Scoping review following Joanna Briggs Institute guidelines and adhering to the PRISMA extension for Scoping Reviews. Searches were carried out in Medline, CINAHL, Web of Science, Cochrane database of systematic reviews, Embase, and APA PsycInfo. Two researchers independently screened all articles published in English or Scandinavian languages and extracted information using a pre-defined template. Refence lists of included articles were screened for additional studies.

**Results:**

Four articles were included, three from psychiatric care and one from substance use disorder treatment. Four different PREMs measures and three generic PROMs measures were used. Each study found PREMs measures to be associated with generic PROMs, but the strength of the associations varied from weak to strong.

**Conclusion:**

Existing studies suggest that patient reported experiences are related to quality of life and well-being among patients in psychiatric and substance use disorder care. This study highlights a critical gap in the understanding of how PREMs and PROMs may interact in these patient populations. Despite limited research on their concurrent use, our findings offer preliminary insights into their potential to support patient-centred care.

## Introduction

Mental health is an essential component of individual health and well-being and an important determinant of social and economic participation and contribution in communities. Poor mental health represents a global, major public health challenge. Currently, more than 1 billion people live with a mental health condition, or a substance use disorder, constituting 7% of the global burden of disease and almost 20% of years lived with disability (1). In Europe, mental health disorders contribute to a substantial share of contacts with health services (2). Although tobacco- and substance use and related mortality has declined globally in recent decades, alcohol- and substance use are hypothesized to play a role in the recent flattening or reversing trends of life expectancy in high-income countries (3).

High quality mental health services should be characterized by effectiveness and safety (4). Moreover, there is a consensus among both politicians, professionals, and patient organizations that mental health care services should be patient-centered, meaning that they should be respectful and responsive to individuals` needs (5). International guidelines on drug use disorders also emphasize the importance of involving patients in service evaluations (6). To deliver such patient-centered care, health care providers need to know how users experience the services they receive. Measurements of patient experiences can be used for several purposes, including to inform policymakers, discussion between service users and health personnel leading to shared decision making, and to guide service quality improvement both at the individual and at the institutional level (7). Clinician reported measures have limited abilities to capture the patients` perspective. Thus, patient reported measures are important to complement other metrics for health care quality. For people with mental health- or substance use disorders, the quality of health care services received is related to health outcomes and related opportunities in life (8). For health care providers, poor quality of services is associated with avoidable, unnecessary use of resources and lost personal contributions (7).

Patient reported experience measures (PREMs) provide patients` view on their experiences with health care services received. Several instruments are developed to measure PREMs in mental health care, covering a range of areas, often including interpersonal relationships, respect and dignity, access and care coordination, drug therapy, information, and care environment (9). The use of PREMs in substance use disorder treatment is also on the rise (10). PREMS have their intrinsic value, but positive patient experiences are also associated with better treatment outcomes including active participation in own care, better communication, adherence to treatment and improved clinical outcomes (8).

Patient reported outcome measures (PROMs) provide information about health outcomes from the patient perspective. PROMs can be specific to symptoms of a certain condition or patient group, or it can be generic, capturing various aspects of subjective wellbeing and of quality of life (5). One commonly used generic PROMs is the WHO-5 wellbeing index, which includes five statements of well-being over the last 2 weeks (11). Other instruments measuring quality of life or well-being are also used, such as Manchester Short Assessment of Quality of Life (MANSA) or Hopkins symptom checklist (11). Generic PROMs can be used over a range of conditions. However, as symptoms of mental health disorders may substantially affect well-being (12), they are especially relevant among patients in psychiatric care and substance use disorder treatment.

PREMs and PROMs are complementary and meant to be used together to provide a more complete picture of how the health care services impact the users (7). The Patient Reported Indicator Surveys (PaRIS) conceptual framework suggest a bi-directional association between PREMS and PROMS inpatients with chronic diseases (13). This means that each can reflect and influence the other, for instance that experiences can impact outcomes or that outcomes may affect experiences. Understanding this relationship fills a significant gap in existing health quality metrics and holds potential for improving long-term outcomes, as these patient populations often require complex, multi-faceted treatment strategies. However, there is little knowledge on how the two types of measures are related among patients in psychiatric care or substance use disorder treatment. There is a need to better understand how the two can work together to support quality improvement in health care services (5) and generate more coordinated care, as these patients have various needs and preferences for care that often require comprehensive and coherently delivered services over time. Understanding the relationship between PREMs and PROMs is therefore crucial for improving patient-centered care and optimizing resource allocation in healthcare settings throughout the service pathway. Integrating patient-reported experiences with outcome measures can lead to more tailored, coordinated and effective treatment strategies. Enhancing the quality of care through better measurement tools is essential for addressing the complexities of mental health and substance use disorders, ultimately leading to better patient outcomes and satisfaction. The aim of this scoping review is thus to assess how generic PROMs have been used in combination with PREMs in mental health care and substance use disorder treatment settings, and to summarize current evidence on how these are related. As we aim to investigate this across patients with various mental health conditions and substance use disorders, generic, and not condition-specific, PROMs were chosen.

## Methods

This scoping review followed the methodology of the Joanna Briggs Institute (14) and we reported according to the Preferred Reporting Items for Systematic Reviews and Meta-Analyses extension for Scoping Reviews (15) (Supplementary Appendix 1). The protocol for the scoping review was published in Open Science Framework (https://osf.io/s7hfj/).

The search strategy was based on a search strategy used in a previous scoping review (16) and further developed by the authors in close collaboration with an experienced librarian at the Norwegian Institute of Public Health. The search strategy, including all identified keywords and index terms, was adapted for each included database (Full search strategy included in Supplementary Appendix 2). The search was carried out in Medline, CINAHL, Web of Science, Cochrane database of systematic reviews, Embase, APA PsycInfo. The final search was carried out on 01.11.2024, with no starting date. The search was limited to articles in English or Scandinavian (Norwegian, Swedish, Danish) languages, based on the language competencies of the research team. We screened reference lists of included articles, as well as of relevant identified systematic reviews, for additional relevant studies. In addition, the websites of selected leading institutions in the field of PREMs, including the Consumer Assessment of Healthcare Providers and Systems (CAHPS) (USA), Picker Institute (England), Netherlands Institute for Health Services Research (NIEVEL), and The National survey of patient experiences (LUP) (Denmark) were searched for relevant publications.

**Figure 1:**
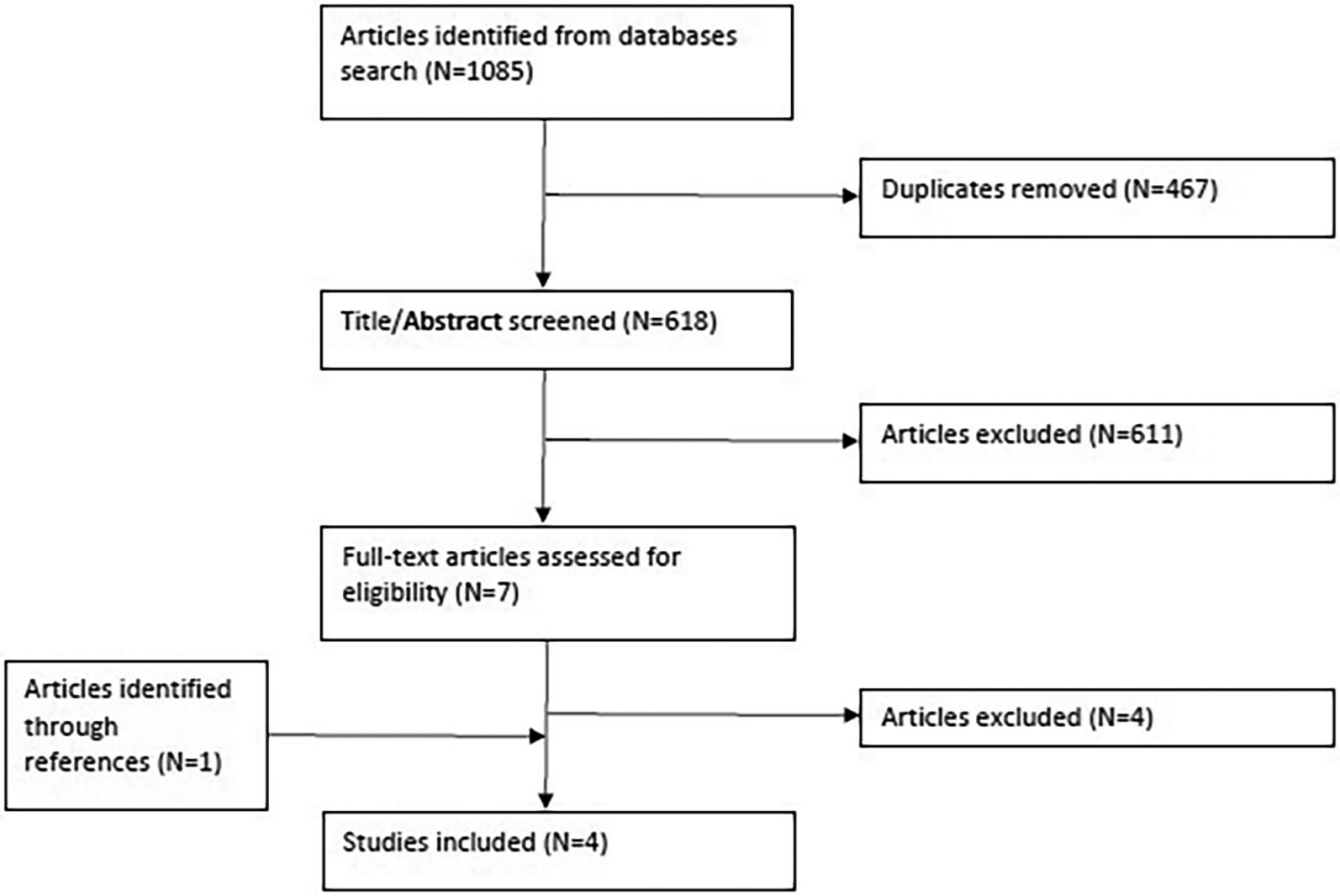
Flow diagram for included studies.

All identified references were uploaded into EndNote 20 and deduplicated. Two authors (MKK, LED) individually screened all titles and abstracts for assessment against the defined inclusion criteria. The same two authors read potentially relevant articles in full text. The two authors had no disagreements in title-, abstract or full-text screening. In full-text screening, the content of each article was discussed. The result of the search and inclusion process is reported in Figure 1. Reasons for exclusions after full-text screening are shown in Supplementary Appendix 3, Supplementary Table 1.

The scoping review includes peer-reviewed quantitative articles reporting on both PREMs and generic PROMs in mental health care or substance use disorder treatment, with a focus on the relationship between the two types of measures. Articles reporting on symptom specific, but not generic, PROMS were not included. We excluded articles reporting from children and adolescent mental health services.

We used an *a priori* extraction form, based on the JBI guidelines for scoping reviews (15) (Supplementary Appendix 4), to systematically extract information from all included studies. Two authors (MKK, LED) independently extracted information from each article to ensure that the information was correct and relevant. The information extraction process followed the pre-defined categories; author, publication year, title, objective, country, context, participants, questionnaire applied for PREMS, domains of experiences assessed, questionnaire applied for PROMs and results regarding the relationship between PREMs and PROMs. Any disagreement between the reviewers regarding extracted information details were resolved through authors discussing and agreeing upon what was of relevance for the scoping review. The extracted information was summarized in tables and a narrative synthesis.

## Results

The database searches resulted in a total of 618 unique articles. After screening of titles and abstracts, seven articles were read in full text. Of these, three were included in the scoping review, together with one additional article found through references screening giving a total of four included studies. The included articles are presented with selected descriptive details in Supplementary Appendix 3, Supplementary Table 2, and summed up in Tables 1, 2.

**Table 1:** Characteristics of included studies.

Characteristic	Number of articles	Reference
Year of publication		
2022	3	(17–19)
2023	1	(5)
Setting		
Inpatient, psychiatric care	2	(5, 18)
Both in-and outpatient psychiatric care	1	(19)
Outpatient substance use disorder treatment	1	(17)
Countries		
USA	1	(17)
Israel	1	(18)
Portugal	1	(19)
France	1	(5)

**Table 2:** PREMs and PROMs instruments used in the included studies.

Instrument type	Name	Dimensions	Reference
PREMs instrument	Set of Questions on Patient Experiences proposed by OECD PaRIS Mental Health Working Group	Courtesy and respect Time spent with the clinician Clarity of the explanations Involvement in decisions about care and treatment	(5, 19)
	Danish national mental health PROM programme (PRO-Psychiatry)	Experience with primary clinician	(18)
	Consumer Assessment of Healthcare Providers and Systems (CAHPS)	Experience with the treatment team: - -being respected - -involved - -receiving sufficient information	(18)
	Communication Quality subscale of the Experiences of Care and Health Outcomes survey (ECHO)	the extent to which patients feel: - -listened to - -respected - -adequately attended to - -that information was conveyed clearly by their providers	(17)
PROMs instrument	The World Health Organization Well-Being Index (WHO-5)	Cheerfulness Calmness Activity Rest Interest	(5, 19)
	The OECD Assessment of Subjective Well-being	Overall life satisfaction Finding meaning and worth in life	(5, 19)
	Manchester Short Assessment of Quality of Life (MANSA)	Quality of life	(18)
	Mental Health component score of the Short-Form-12 (MCS-12)	Overall emotional well-being	(17)

### Description of included studies

The four included studies were carried out in four different countries, USA (17), Israel (18), Portugal (19) and France (5) (Table 1). Three articles were published in 2022 (17–19) and one in 2023 (5). The studies were conducted in both inpatient- (5, 18) and outpatient settings (17) or both (19).

### PREMs and PROMs instruments

The studies used various instruments to capture PREMS and PROMS. Two studies used the PREMS instrument suggested by the OECD PaRIS Mental Health Working Group (5, 19). One study used an instrument developed by the Danish national mental health programme, together with the CAHPS instrument (18), and one used the Communication Quality subscale of the Experiences of Care and Health Outcomes survey (ECHO) instrument (17). The dimensions of care covered by the instruments are described in Table 2. In the studies which described the content of their instruments, the dimensions included were to a large degree overlapping, including respect, involvement, and information. How well the focus of the included PREMs instruments (or parts of the instruments) was described varied between the studies.

Regarding generic PROMs instruments, two studies used the World Health Organization Well-Being Index (WHO-5) and two additional questions on life satisfaction and finding meaning in life, as recommended by OECD (5, 19) (Table 2). One study used the Manchester Short Assessment of Quality of Life (MANSA) (18) and one used the Mental Health component score of the Short-Form-12 (MCS-12) (17).

### Synthesis of evidence

All included studies assessed associations between PREMS measures and generic PROMs measures among patients in psychiatric care or substance use disorder treatment. Each study found PREMs measures to be associated with generic PROMs, but the strength of the associations varied from weak to strong. Among inpatients, strong associations between two different PREMs instruments and quality of life were found in Israel (18), whereas a moderate, and non-significant, association between PREMs and overall well-being were reported in a French study (5). In a sample consisting of mainly outpatients in Portugal (19), a significant association between PREMs and PROMs measures was found, whereas Liebmann et al. (17) found that problems with communication and overall satisfaction were both related to slightly lower scores of overall emotional well-being among Veterans in outpatient clinics in the US.

## Discussion

Few studies were found reporting on the relationship between PREMs and PROMs in psychiatric care and substance use disorder treatment. This study therefore reveals a knowledge gap regarding the use of PREMs and PROMs, which means we lack information that could support more tailored, coordinated, and effective services for these patient groups. The few existing studies on PREMs and PROMs integration in mental health and substance use disorder treatment underscore the novelty of this research. Establishing clearer links between patient experiences and health outcomes in this context is essential for fostering better-coordinated and outcome-driven services. Three studies from psychiatric care and one study from substance use disorder treatment were included. All studies indicate a relationship between higher scores on both PREMs and PROMs measures in both inpatient and outpatient settings, but the strength of the associations varied across studies.

The importance of implementing PREMs and PROMs measurements in mental health care to achieve people-centered care has been highlighted in recent years (7). However, there has been little focus on the relationship between them and how to use them together. Although PREMs and PROMs are extensively used in monitoring the quality of mental health care (9, 20), and several actors collect both measures, few studies have assessed the association between these measures, as evident from the literature search in the current article. The fact that all included articles were published within the two last years, may indicate that the interest and emphasis on the topic is on the rise.

Patient satisfaction was included in the PREMs measures in two studies (5, 17). Patient experiences include, but is not limited to, patient satisfaction. Patient satisfaction relates to a patient`s subjective evaluation of the services, whereas patient experiences relate to more objective information about services received. Patient satisfaction is dependent on a person`s standards and expectations (21). In quality development work, patient experiences may be the most informative.

The included articles all indicate that generic PROMs measures, either subjective well-being or quality of life, are related to the experiences patients have in mental health care and substance use disorder treatment. This is in line with previous studies in other patient groups, showing associations between patient reported experiences and measures of quality of life and subjective well-being (8, 22–23). Whereas weaker associations have been seen between patient experiences and generic PROMs measures than between patient experiences and symptom specific PROMs among patients in elective surgery (22), Mendlovic et al. found a stronger relationship between patient experiences and generic PROMs measures than to symptom-specific PROMs among patients in psychiatric care (18). They argue that patients may experience hope and high quality of life, despite symptoms of disease, and that their experiences in care may be involved in this. It might be that being seen, heard, and respected in care can improve onès quality of life. As the studies in this scoping review are all cross-sectional, we cannot conclude whether patients who rate their subjective wellbeing or quality of life best are more likely to have positive experiences in care, or conversely, that the experiences in care are determinants of well-being and quality of life. No matter which way the association goes, this indicates the importance of measuring and interpreting the PREMs and generic PROMs measures together. Previous studies have shown that evaluations of well-being differ between patients and care providers (24), but it is suggested that use of PROMS instruments can help bridge this gap between the ratings (19, 24).

Future research should focus on longitudinal studies to better understand the causal relationships between PREMs and PROMs. Additionally, exploring the impact of sociodemographic factors on these relationships can provide insights into how different patient groups perceive and benefit from mental health and substance use disorder treatments. This may lead to more personalized care approaches tailored to the diverse needs of various patient populations.

### Strengths and limitations

The scoping review has several methodological strengths. The search was carried out in six databases covering medicine, mental health literature and social science. Guidelines from the Joanna Briggs Institute were followed, and we adhered to the Preferred Reporting Items for Systematic Reviews and Meta-Analyses extension for Scoping Review.

A limited number of relevant studies were identified. All studies were from different countries, represented both inpatient and outpatient settings and both psychiatric care and substance use disorder treatment. As many with substance use disorders also have a mental health condition, we anticipate that findings from one of the settings may be relevant for the other. Moreover, the mode of data collection and mental health diagnoses varied across studies. This makes it difficult to draw firm conclusions from the scoping review. We included articles published in English only (none found in Scandinavian languages) and may have missed relevant studies published in other languages. However, we assume that most relevant literature is published in English. We did not perform a quality assessment of each paper.

### Implications

As the number of studies reporting on associations between PREMs and generic PROMs in mental health care and substance use disorder treatment is still small, further research investigating this relationship is warranted. Further studies should assess the unique specificity of various measures of generic PROMs, how PREMs and PROMs are related in distinct mental health care and substance use disorder treatment settings, and differences in associations by sociodemographic variables. Such studies are needed to gain insight into how to best utilize measurements of PREMs and PROMs together in quality improvement work and to support shared decision-making and patient-centred care. As patient experiences seem to be related to quality of life, it will be important in the future to also assess generic PROMs measures in relation to follow-up of patients after hospital discharge and in further coordination of care. Our findings indicate that a more systematic use of PREMs and PROMs could allow health services to better track and address evolving patient needs. Given the importance of monitoring the wellbeing of these vulnerable patient groups over time, this research provides valuable groundwork for future longitudinal studies.

## Conclusion

The few existing studies suggest that patient reported experiences with care are related to quality of life and well-being among patients in psychiatric care and substance use disorder treatment. Understanding and utilizing these relationships has the potential to greatly enhance patient-centred care, leading to improved health outcomes and patient satisfaction.
