# Supplementary material for: Concurrent use and association of patient-reported experience and outcome measures in psychiatric and substance use disorder care: a scoping review
Source: Front Health Serv. 2025 Jun 30;5:1620809. doi: 10.3389/frhs.2025.1620809 (PMC12256552; doi:10.3389/frhs.2025.1620809)
Supplement: Supplementary file 2 [file Table2.docx]

## Appendix 2

The initial search was conducted 25.12.2023. The search was updated November 2024 to include hits from 25.12.2023 to 01.11.2024, see below for results from the updated search.

## Search strategy

**Database: Ovid MEDLINE(R) and Epub Ahead of Print, In-Process, In-Data-Review & Other Non-Indexed Citations, Daily and Versions 1946 to December 22, 2023**

**Date:** 25.12.2023

**Number of hits:** 183

| 1 | Residential Treatment/ | 3336 |
| --- | --- | --- |
| 2 | exp Mental Disorders/ or exp Substance-Related Disorders/ | 1484499 |
| 3 | 1 and 2 | 2558 |
| 4 | Psychiatric department, Hospital/ or Hospitals, Psychiatric/ or Emergency Services, Psychiatric/ or Therapeutic Community/ | 36391 |
| 5 | ("speciali#ed mental health service?" or therapeutic communit* or ((mental or psychiatric) adj3 (inpatient? or ward? or department? or unit? or hospital* or institution? or primary health care or primary care or community care or residential care or "residential treatment?")) or (emergency adj1 psychiatric adj1 service?)).tw,kf. | 47385 |
| 6 | Substance Abuse Treatment Centers/ | 5426 |
| 7 | ((drug or substance or addiction or dependence or alcohol* or opioid? or opiate) adj3 ("inpatient clinic?" or center? or centre? or ward? or unit? or department? or rehab* or treatment? or therap* or intervention? or primary health care or primary care or community care or residential care)).tw,kf. | 238250 |
| 8 | or/3-7 | 306020 |
| 9 | Patient Satisfaction/ | 90050 |
| 10 | (((consumer? or patient? or user? or client? or care or healthcare or service or treatment) adj3 (experience* or satisfaction or assessment* or evaluat* or rating* or opinion* or judg* or perception* or perceive? or perspective* or "point-of-view")) or (("patient reported" or "self reported" or patientreported or selfreported) adj (satisfaction or experience* or perception? or preference*)) or PREM or PREMs).tw,kf. | 768550 |
| 11 | or/9-10 | 812688 |
| 12 | Patient Reported Outcome Measures/ | 14292 |
| 13 | (((consumer? or patient? or user? or client? or care or healthcare or "health care" or service or treatment) adj3 (("patient reported" or "self reported" or patientreported or selfreported) adj (outcome* or result?))) or PROM or PROMs).tw,kf. | 42945 |
| 14 | or/12-13 | 46250 |
| 15 | 8 and 11 and 14 | 183 |

**Database: Embase 1974 to 2023 December 22**

**Date:** 25.12.2023

**Number of hits:** 352

| 1 | residential care/ | 13589 |
| --- | --- | --- |
| 2 | exp mental disease/ or substance abuse/ or alcohol abuse/ | 2775333 |
| 3 | 1 and 2 | 5825 |
| 4 | psychiatric department/ or therapeutic community/ | 11710 |
| 5 | ("speciali#ed mental health service?" or therapeutic communit* or ((mental or psychiatric) adj3 (inpatient? or ward? or department? or unit? or hospital* or institution? or primary health care or primary care or community care or residential care or "residential treatment?")) or (emergency adj1 psychiatric adj1 service?)).tw,kf. | 58919 |
| 6 | drug dependence treatment/ or alcohol rehabilitation program/ | 9714 |
| 7 | ((drug or substance or addiction or dependence or alcohol* or opioid? or opiate) adj3 ("inpatient clinic?" or center? or centre? or ward? or unit? or department? or rehab* or treatment? or therap* or intervention? or primary health care or primary care or community care or residential care)).tw,kf. | 326999 |
| 8 | or/3-7 | 394734 |
| 9 | patient satisfaction/ | 172812 |
| 10 | (((consumer? or patient? or user? or client? or care or healthcare or service or treatment) adj3 (experience* or satisfaction or assessment* or evaluat* or rating* or opinion* or judg* or perception* or perceive? or perspective* or "point-of-view")) or (("patient reported" or "self reported" or patientreported or selfreported) adj (satisfaction or experience* or perception? or preference*)) or PREM or PREMs).tw,kf. | 1192243 |
| 11 | or/9-10 | 1271951 |
| 12 | patient-reported outcome/ | 56973 |
| 13 | (((consumer? or patient? or user? or client? or care or healthcare or "health care" or service or treatment) adj3 (("patient reported" or "self reported" or patientreported or selfreported) adj (outcome* or result?))) or PROM or PROMs).tw,kf. | 69746 |
| 14 | or/12-13 | 85127 |
| 15 | 8 and 11 and 14 | 380 |
| 16 | limit 15 to (conference abstracts or embase or "preprints (unpublished, non-peer reviewed)") | 352 |

**Database: APA PsycInfo 1806 to December Week 2 2023**

**Date:** 25.12.2023

**Number of hits:** 52

| 1 | Psychiatric units/ or Psychiatric Hospitals/ or Psychiatric hospitalization/ or Therapeutic Community/ | 20728 |
| --- | --- | --- |
| 2 | ("speciali#ed mental health service?" or therapeutic communit* or ((mental or psychiatric) adj3 (inpatient? or ward? or department? or unit? or hospital* or institution? or primary health care or primary care or community care or residential care or "residential treatment?")) or (emergency adj1 psychiatric adj1 service?)).tw. | 54047 |
| 3 | Drug Rehabilitation/ or Alcohol Rehabilitation/ | 31340 |
| 4 | ((drug or substance or addiction or dependence or alcohol* or opioid? or opiate) adj3 ("inpatient clinic?" or center? or centre? or ward? or unit? or department? or rehab* or treatment? or therap* or intervention? or primary health care or primary care or community care or residential care)).tw. | 84998 |
| 5 | or/1-4 | 151051 |
| 6 | Client Satisfaction/ | 6552 |
| 7 | (((consumer? or patient? or user? or client? or care or healthcare or service or treatment) adj3 (experience* or satisfaction or assessment* or evaluat* or rating* or opinion* or judg* or perception* or perceive? or perspective* or "point-of-view")) or (("patient reported" or "self reported" or patientreported or selfreported) adj (satisfaction or experience* or perception? or preference*)) or PREM or PREMs).tw. | 189005 |
| 8 | or/6-7 | 189933 |
| 9 | Patient Reported Outcome Measures/ | 830 |
| 10 | (((consumer? or patient? or user? or client? or care or healthcare or "health care" or service or treatment) adj3 (("patient reported" or "self reported" or patientreported or selfreported) adj (outcome* or result?))) or PROM or PROMs).tw. | 4320 |
| 11 | or/9-10 | 4392 |
| 12 | 5 and 8 and 11 | 52 |

**Database: Web of Science Core Collection: Science Citation Index Expanded (SCI-EXPANDED) --1987-present, Social Sciences Citation Index (SSCI) --1987-present, Arts & Humanities Citation Index (A&HCI) --1987-present, Emerging Sources Citation Index (ESCI) --2015-present**

**Date:** 25.12.2023

**Number of hits:** 188

| 1 | TS=(("speciali?ed mental health service$" OR "therapeutic communit*" OR ((mental OR psychiatric ) NEAR/2 ("inpatient clinic$" OR "inpatient psychiatric" OR ward$ OR department$ OR unit$ OR hospital* OR institution$ OR "primary health care" OR "primary care" OR "community care" OR "residential care" OR "residential treatment$" )) OR (emergency NEAR/0 psychiatric NEAR/0 service$ ))) | 32088 |
| --- | --- | --- |
| 2 | TS=(((drug OR substance OR addiction OR dependence OR alcohol* OR opioid$ OR opiate ) NEAR/2 ("inpatient clinic$" OR center$ OR centre$ OR ward$ OR unit$ OR department$ OR rehab* OR treatment$ OR therap* OR intervention$ OR "primary health care" OR "primary care" OR "community care" OR "residential care" ))) | 265009 |
| 3 | #1 OR #2 | 295250 |
| 4 | TS=((((consumer$ OR patient$ OR user$ OR client$ OR care OR healthcare OR service OR treatment ) NEAR/2 (experience* OR satisfaction OR assessment* OR evaluat* OR rating* OR opinion* OR judg* OR perception* OR perceive$ OR perspective* OR "point-of-view")) OR (("patient reported" OR "self reported" OR patientreported OR selfreported ) NEAR/0 (satisfaction OR experience* OR perception$ OR preference* )) OR PREM OR PREMs )) | 795899 |
| 5 | TS=((((consumer$ OR patient$ OR user$ OR client$ OR care OR healthcare OR " health care" OR service OR treatment ) NEAR/2 (("patient reported" OR "self reported" OR patientreported OR selfreported ) NEAR/0 (outcome* OR result$ ))) OR PROM OR PROMs ) | 49457 |
| 6 | #3 AND #4 AND #5 | 188 |

**Database: Cochrane Database of Systematic Reviews:** **Issue 12 of 12, December 2023**2023**Central Register of Controlled trials: Issue 11 of 12, November 2023**

**Date:** 07.12.2023

**NUmber of hits:** 3 (SR); 95(fra trials)

| ID | Search | Hits |
| --- | --- | --- |
| #1 | [mh ^"Residential Treatment"] | 199 |
| #2 | [mh "Mental Disorders"] | 100688 |
| #3 | [mh "Substance-Related Disorders"] | 19950 |
| #4 | #2 or #3 | 101500 |
| #5 | #1 and #4 | 182 |
| #6 | [mh ^"Psychiatric department, Hospital"] | 112 |
| #7 | [mh ^"Hospitals, Psychiatric"] | 288 |
| #8 | [mh ^"Emergency Services, Psychiatric"] | 56 |
| #9 | [mh ^"Therapeutic Community"] | 67 |
| #10 | ((("specialized mental health" or "specialised mental health") NEXT service?) or (therapeutic NEXT communit*) or ((mental or psychiatric) NEAR/3 (inpatient? or ward? or department? or unit? or hospital* or institution? or "primary health care" or "primary care" or "community care" or "residential care" or "residential treatment" or "residential treatments")) or asylum or (("emergency psychiatric" or "psychiatric emergency") NEAR/1 service?)):ti,ab | 3786 |
| #11 | [mh ^"Substance Abuse Treatment Centers"] | 406 |
| #12 | ((drug or substance or addiction or dependence or alcohol* or opioid? or opiate) NEAR/3 ("inpatient clinic" or "inpatient clinics" or center? or centre? or ward? or unit? or department? or rehab* or treatment? or therap* or intervention? or "primary health care" or "primary care" or "community care" or "residential care")):ti,ab | 43039 |
| #13 | {or #5-#12} | 46485 |
| #14 | [mh ^"Patient Satisfaction"] | 14816 |
| #15 | (((consumer? OR patient? OR user? OR client? OR care OR healthcare OR " health care" OR service OR treatment) NEAR/3 (experience* OR satisfaction OR assessment* OR evaluat* OR rating* OR opinion* OR judg* OR perception* OR perceive? OR perspective* OR point-of-view)) OR (("patient reported" OR "self reported" OR patientreported OR selfreported ) NEXT (satisfaction OR experience* OR perception? OR preference* )) OR PREM OR PREMs ):ti,ab | 147199 |
| #16 | #14 OR #15 | 154043 |
| #17 | [mh ^"Patient Reported Outcome Measures"] | 1393 |
| #18 | (((consumer? OR patient? OR user? OR client? OR care OR healthcare OR "health care" OR service OR treatment ) NEAR/3 (("patient reported" OR "self reported" OR patientreported OR selfreported ) NEXT (outcome* OR result? ))) OR PROM OR PROMs ):ti,ab | 14137 |
| #19 | #17 OR #18 | 14137 |
| #20 | #13 and #16 and #19 | 98 |

**Database: CINAHL (EBSCO)**

**Date:** 08.12.2023

**Number of hits:** 30

| **#** | **Query** | **Results** |
| --- | --- | --- |
| S1 | (MH "Hospitals, Psychiatric") or (MH "Psychiatric units") or (MH "Community Mental Health Nursing") | 11,344 |
| S2 | TI ( ("speciali?ed mental health service#" OR "therapeutic communit*" OR ((mental OR psychiatric ) N2 ("inpatient clinic#" OR "inpatient psychiatric" OR ward# OR department# OR unit# OR hospital* OR institution# OR "primary health care" OR "primary care" OR "community care" OR "residential care" OR "residential treatment#" )) OR (emergency N0 psychiatric N0 service# )) ) OR AB ( ("speciali?ed mental health service#" OR "therapeutic communit*" OR ((mental OR psychiatric ) N2 ("inpatient clinic#" OR "inpatient psychiatric" OR ward# OR department# OR unit# OR hospital* OR institution# OR "primary health care" OR "primary care" OR "community care" OR "residential care" OR "residential treatment#" )) OR (emergency N0 psychiatric N0 service# )) ) | 16,218 |
| S3 | (MH "Substance Use Rehabilitation Programs+") | 14,216 |
| S4 | TI ( ((drug OR substance OR addiction OR dependence OR alcohol* OR opioid# OR opiate ) N2 ("inpatient clinic#" OR center# OR centre# OR ward# OR unit# OR department# OR rehab* OR treatment# OR therap* OR intervention# OR "primary health care" OR "primary care" OR "community care" OR "residential care" )) ) OR AB ( ((drug OR substance OR addiction OR dependence OR alcohol* OR opioid# OR opiate ) N2 ("inpatient clinic#" OR center# OR centre# OR ward# OR unit# OR department# OR rehab* OR treatment# OR therap* OR intervention# OR "primary health care" OR "primary care" OR "community care" OR "residential care" )) ) | 67,161 |
| S5 | S1 OR S2 OR S3 OR S4 | 99,077 |
| S6 | TI ( (((consumer# OR patient# OR user# OR client# OR care OR healthcare OR " health care" OR service OR treatment ) N2 (experience* OR satisfaction OR assessment* OR evaluat* OR rating* OR opinion* OR judg* OR perception* OR perceive# OR perspective* OR point-of-view )) OR (("patient reported" OR "self reported" OR patientreported OR selfreported ) W0 (satisfaction OR experience* OR perception# OR preference* )) OR PREM OR PREMs ) ) OR AB ( (((consumer# OR patient# OR user# OR client# OR care OR healthcare OR " health care" OR service OR treatment ) N2 (experience* OR satisfaction OR assessment* OR evaluat* OR rating* OR opinion* OR judg* OR perception* OR perceive# OR perspective* OR point-of-view )) OR (("patient reported" OR "self reported" OR patientreported OR selfreported ) W0 (satisfaction OR experience* OR perception# OR preference* )) OR PREM OR PREMs ) ) | 294,277 |
| S7 | (MH "Patient Satisfaction") | 62,176 |
| S8 | S6 OR S7 | 329,142 |
| S9 | (MH "Patient-Reported Outcomes") | 6,975 |
| S10 | TI ( (((consumer# OR patient# OR user# OR client# OR care OR healthcare OR "health care" OR service OR treatment ) N2 (("patient reported" OR "self reported" OR patientreported OR selfreported ) W0 (outcome* OR result# ))) OR PROM OR PROMs ) ) ) OR AB ( (((consumer# OR patient# OR user# OR client# OR care OR healthcare OR "health care" OR service OR treatment ) N2 (("patient reported" OR "self reported" OR patientreported OR selfreported ) W0 (outcome* OR result# ))) OR PROM OR PROMs ) ) ) | 17,840 |
| S11 | S9 OR S10 | 20,849 |
| S12 | S5 AND S8 AND S11 | 75 |

**Database: Ovid MEDLINE(R) and Epub Ahead of Print, In-Process, In-Data-Review & Other Non-Indexed Citations, Daily and Versions 1946 to November 05, 2024**

**Date:** 01.11.2024

**Number of hits:** 33

| 1 | Residential Treatment/ | 3395 |
| --- | --- | --- |
| 2 | exp Mental Disorders/ or exp Substance-Related Disorders/ | 1535575 |
| 3 | 1 and 2 | 2612 |
| 4 | Psychiatric department, Hospital/ or Hospitals, Psychiatric/ or Emergency Services, Psychiatric/ or Therapeutic Community/ | 36647 |
| 5 | ("speciali#ed mental health service?" or therapeutic communit* or ((mental or psychiatric) adj3 (inpatient? or ward? or department? or unit? or hospital* or institution? or primary health care or primary care or community care or residential care or "residential treatment?")) or (emergency adj1 psychiatric adj1 service?)).tw,kf. | 49071 |
| 6 | Substance Abuse Treatment Centers/ | 5468 |
| 7 | ((drug or substance or addiction or dependence or alcohol* or opioid? or opiate) adj3 ("inpatient clinic?" or center? or centre? or ward? or unit? or department? or rehab* or treatment? or therap* or intervention? or primary health care or primary care or community care or residential care)).tw,kf. | 249906 |
| 8 | or/3-7 | 319359 |
| 9 | Patient Satisfaction/ | 92582 |
| 10 | (((consumer? or patient? or user? or client? or care or healthcare or service or treatment) adj3 (experience* or satisfaction or assessment* or evaluat* or rating* or opinion* or judg* or perception* or perceive? or perspective* or "point-of-view")) or (("patient reported" or "self reported" or patientreported or selfreported) adj (satisfaction or experience* or perception? or preference*)) or PREM or PREMs).tw,kf. | 819787 |
| 11 | or/9-10 | 864392 |
| 12 | Patient Reported Outcome Measures/ | 16630 |
| 13 | (((consumer? or patient? or user? or client? or care or healthcare or "health care" or service? or treatment?) adj3 (("patient reported" or "self reported" or patientreported or selfreported) adj (outcome* or result?))) or PROM or PROMs).tw,kf. | 49262 |
| 14 | or/12-13 | 52827 |
| 15 | 8 and 11 and 14 | 211 |
| 16 | 2024*.ed,ep,yr,dp,dt. | 1580640 |
| 17 | 202312*.ep,ed,dt. | 200310 |
| 18 | or/16-17 | 1685742 |
| 19 | 15 and 18 | 33 |

**Database: Embase 1974 to 2024 November 05**

**Dato:** 01.11.2024

**Number of hits:** 56

| 1 | residential care/ | 14053 |
| --- | --- | --- |
| 2 | exp mental disease/ or substance abuse/ or alcohol abuse/ | 2928806 |
| 3 | 1 and 2 | 6110 |
| 4 | psychiatric department/ or therapeutic community/ | 12244 |
| 5 | ("speciali#ed mental health service?" or therapeutic communit* or ((mental or psychiatric) adj3 (inpatient? or ward? or department? or unit? or hospital* or institution? or primary health care or primary care or community care or residential care or "residential treatment?")) or (emergency adj1 psychiatric adj1 service?)).tw,kf. | 61839 |
| 6 | drug dependence treatment/ or alcohol rehabilitation program/ | 9890 |
| 7 | ((drug or substance or addiction or dependence or alcohol* or opioid? or opiate) adj3 ("inpatient clinic?" or center? or centre? or ward? or unit? or department? or rehab* or treatment? or therap* or intervention? or primary health care or primary care or community care or residential care)).tw,kf. | 343990 |
| 8 | or/3-7 | 414918 |
| 9 | patient satisfaction/ | 182829 |
| 10 | (((consumer? or patient? or user? or client? or care or healthcare or service or treatment) adj3 (experience* or satisfaction or assessment* or evaluat* or rating* or opinion* or judg* or perception* or perceive? or perspective* or "point-of-view")) or (("patient reported" or "self reported" or patientreported or selfreported) adj (satisfaction or experience* or perception? or preference*)) or PREM or PREMs).tw,kf. | 1270291 |
| 11 | or/9-10 | 1353696 |
| 12 | patient-reported outcome/ | 67109 |
| 13 | (((consumer? or patient? or user? or client? or care or healthcare or "health care" or service? or treatment?) adj3 (("patient reported" or "self reported" or patientreported or selfreported) adj (outcome* or result?))) or PROM or PROMs).tw,kf. | 82417 |
| 14 | or/12-13 | 100193 |
| 15 | 8 and 11 and 14 | 446 |
| 16 | 2024*.yr,dd,dp,dc. | 1824613 |
| 17 | 202312*.dd,dc. | 155979 |
| 18 | or/16-17 | 1956277 |
| 19 | 15 and 18 | 50 |

**Database: APA PsycInfo 1806 to October 2024 Week 5**

**Date:** 01.11.2024

**Number of hits:** 12

| 1 | exp Residential Care Institutions/ | 52606 |
| --- | --- | --- |
| 2 | exp Mental Disorders/ or drug abuse/ | 1144758 |
| 3 | 1 and 2 | 15145 |
| 4 | Psychiatric units/ or Psychiatric Hospitals/ or Psychiatric hospitalization/ or Therapeutic Community/ | 21293 |
| 5 | ("speciali#ed mental health service?" or therapeutic communit* or ((mental or psychiatric) adj3 (inpatient? or ward? or department? or unit? or hospital* or institution? or primary health care or primary care or community care or residential care or "residential treatment?")) or (emergency adj1 psychiatric adj1 service?)).tw. | 55466 |
| 6 | Drug Rehabilitation/ or Alcohol Rehabilitation/ | 32385 |
| 7 | ((drug or substance or addiction or dependence or alcohol* or opioid? or opiate) adj3 ("inpatient clinic?" or center? or centre? or ward? or unit? or department? or rehab* or treatment? or therap* or intervention? or primary health care or primary care or community care or residential care)).tw. | 87252 |
| 8 | or/3-7 | 164917 |
| 9 | Client Satisfaction/ | 6899 |
| 10 | (((consumer? or patient? or user? or client? or care or healthcare or service or treatment) adj3 (experience* or satisfaction or assessment* or evaluat* or rating* or opinion* or judg* or perception* or perceive? or perspective* or "point-of-view")) or (("patient reported" or "self reported" or patientreported or selfreported) adj (satisfaction or experience* or perception? or preference*)) or PREM or PREMs).tw. | 199045 |
| 11 | or/9-10 | 200017 |
| 12 | Patient Reported Outcome Measures/ | 1012 |
| 13 | (((consumer? or patient? or user? or client? or care or healthcare or "health care" or service or treatment) adj3 (("patient reported" or "self reported" or patientreported or selfreported) adj (outcome* or result?))) or PROM or PROMs).tw. | 4824 |
| 14 | or/12-13 | 4917 |
| 15 | 8 and 11 and 14 | 65 |
| 16 | 2024*.yr,dp,up. | 194890 |
| 17 | 202312*.up. | 12634 |
| 18 | or/16-17 | 203537 |
| 19 | 15 and 18 | 12 |

**Database: Web of Science Core Collection: Science Citation Index Expanded (SCI-EXPANDED) --1987-present, Social Sciences Citation Index (SSCI) --1987-present, Arts & Humanities Citation Index (A&HCI) --1987-present, Emerging Sources Citation Index (ESCI) --2015-present**

**Date:** 06.11.2024

**Number of hits:** 20

| 1 | TS=(("speciali?ed mental health service$" OR "therapeutic communit*" OR ((mental OR psychiatric ) NEAR/2 ("inpatient clinic$" OR "inpatient psychiatric" OR ward$ OR department$ OR unit$ OR hospital* OR institution$ OR "primary health care" OR "primary care" OR "community care" OR "residential care" OR "residential treatment$" )) OR (emergency NEAR/0 psychiatric NEAR/0 service$ ))) | 34303 |
| --- | --- | --- |
| 2 | TS=(((drug OR substance OR addiction OR dependence OR alcohol* OR opioid$ OR opiate ) NEAR/2 ("inpatient clinic$" OR center$ OR centre$ OR ward$ OR unit$ OR department$ OR rehab* OR treatment$ OR therap* OR intervention$ OR "primary health care" OR "primary care" OR "community care" OR "residential care" ))) | 233972 |
| 3 | #1 OR #2 | 266421 |
| 4 | TS=((((consumer$ OR patient$ OR user$ OR client$ OR care OR healthcare OR service OR treatment ) NEAR/2 (experience* OR satisfaction OR assessment* OR evaluat* OR rating* OR opinion* OR judg* OR perception* OR perceive$ OR perspective* OR "point-of-view")) OR (("patient reported" OR "self reported" OR patientreported OR selfreported ) NEAR/0 (satisfaction OR experience* OR perception$ OR preference* )) OR PREM OR PREMs )) | 841219 |
| 5 | TS=(((consumer$ OR patient$ OR user$ OR client$ OR care OR healthcare OR " health care" OR service OR treatment ) NEAR/2 (("patient reported" OR "self reported" OR patientreported OR selfreported ) NEAR/0 (outcome* OR result$ ))) OR PROM OR PROMs ) | 57466 |
| 6 | #3 AND #4 AND #5 | 191 |
| 7 | #6 Timespan: 2023-12-01 to 2024-10-06 | 20 |

**Database: Cochrane Database of Systematic Reviews:** **Issue 12 of 12, December 2023**2023**Central Register of Controlled trialsIssue 11 of 12, November 2024**

**Dato:** 01.11.2024

**Number of hits:** 0(SR); 9(from trials)

| #1 | [mh ^"Residential Treatment"] | 220 |
| --- | --- | --- |
| #2 | [mh "Mental Disorders"] | 109219 |
| #3 | [mh "Substance-Related Disorders"] | 21447 |
| #4 | #2 or #3 | 109869 |
| #5 | #1 and #4 | 197 |
| #6 | [mh ^"Psychiatric department, Hospital"] | 133 |
| #7 | [mh ^"Hospitals, Psychiatric"] | 306 |
| #8 | [mh ^"Emergency Services, Psychiatric"] | 63 |
| #9 | [mh ^"Therapeutic Community"] | 65 |
| #10 | ((("specialized mental health" or "specialised mental health") NEXT service?) or (therapeutic NEXT communit*) or ((mental or psychiatric) NEAR/3 (inpatient? or ward? or department? or unit? or hospital* or institution? or "primary health care" or "primary care" or "community care" or "residential care" or "residential treatment" or "residential treatments")) or (("emergency psychiatric" or "psychiatric emergency") NEAR/1 service?)):ti,ab | 4029 |
| #11 | [mh ^"Substance Abuse Treatment Centers"] | 422 |
| #12 | ((drug or substance or addiction or dependence or alcohol* or opioid? or opiate) NEAR/3 ("inpatient clinic" or "inpatient clinics" or center? or centre? or ward? or unit? or department? or rehab* or treatment? or therap* or intervention? or "primary health care" or "primary care" or "community care" or "residential care")):ti,ab | 45820 |
| #13 | {or #5-#12} | 49967 |
| #14 | [mh ^"Patient Satisfaction"] | 14695 |
| #15 | (((consumer? OR patient? OR user? OR client? OR care OR healthcare OR " health care" OR service OR treatment) NEAR/3 (experience* OR satisfaction OR assessment* OR evaluat* OR rating* OR opinion* OR judg* OR perception* OR perceive? OR perspective* OR point-of-view)) OR (("patient reported" OR "self reported" OR patientreported OR selfreported ) NEXT (satisfaction OR experience* OR perception? OR preference* )) OR PREM OR PREMs ):ti,ab | 160420 |
| #16 | #14 OR #15 | 167305 |
| #17 | [mh ^"Patient Reported Outcome Measures"] | 1939 |
| #18 | (((consumer? OR patient? OR user? OR client? OR care OR healthcare OR "health care" OR service OR treatment ) NEAR/3 (("patient reported" OR "self reported" OR patientreported OR selfreported ) NEXT (outcome* OR result? ))) OR PROM OR PROMs ):ti,ab | 15954 |
| #19 | #17 OR #18 | 16502 |
| #20 | #13 and #16 and #19 with Cochrane Library publication date Between Dec 2023 and Nov 2024, in Cochrane Reviews, Trials | 9 |

**Database: CINAHL (EBSCO)**

**Date:** 01.11.2024

**Number of hits:** 22

| **#** | **Query** | **Results** |
| --- | --- | --- |
| S1 | (MH "Hospitals, Psychiatric") or (MH "Psychiatric units") or (MH "Community Mental Health Nursing") | 11,780 |
| S2 | TI ( ("speciali?ed mental health service#" OR "therapeutic communit*" OR ((mental OR psychiatric ) N2 ("inpatient clinic#" OR "inpatient psychiatric" OR ward# OR department# OR unit# OR hospital* OR institution# OR "primary health care" OR "primary care" OR "community care" OR "residential care" OR "residential treatment#" )) OR (emergency N0 psychiatric N0 service# )) ) OR AB ( ("speciali?ed mental health service#" OR "therapeutic communit*" OR ((mental OR psychiatric ) N2 ("inpatient clinic#" OR "inpatient psychiatric" OR ward# OR department# OR unit# OR hospital* OR institution# OR "primary health care" OR "primary care" OR "community care" OR "residential care" OR "residential treatment#" )) OR (emergency N0 psychiatric N0 service# )) ) | 16,085 |
| S3 | (MH "Substance Use Rehabilitation Programs+") | 14,196 |
| S4 | TI ( ((drug OR substance OR addiction OR dependence OR alcohol* OR opioid# OR opiate ) N2 ("inpatient clinic#" OR center# OR centre# OR ward# OR unit# OR department# OR rehab* OR treatment# OR therap* OR intervention# OR "primary health care" OR "primary care" OR "community care" OR "residential care" )) ) OR AB ( ((drug OR substance OR addiction OR dependence OR alcohol* OR opioid# OR opiate ) N2 ("inpatient clinic#" OR center# OR centre# OR ward# OR unit# OR department# OR rehab* OR treatment# OR therap* OR intervention# OR "primary health care" OR "primary care" OR "community care" OR "residential care" )) ) | 65,765 |
| S5 | S1 OR S2 OR S3 OR S4 | 98,314 |
| S6 | TI ( (((consumer# OR patient# OR user# OR client# OR care OR healthcare OR " health care" OR service OR treatment ) N2 (experience* OR satisfaction OR assessment* OR evaluat* OR rating* OR opinion* OR judg* OR perception* OR perceive# OR perspective* OR "point-of-view")) OR (("patient reported" OR "self reported" OR patientreported OR selfreported ) W0 (satisfaction OR experience* OR perception# OR preference* )) OR PREM OR PREMs ) ) OR AB ( (((consumer# OR patient# OR user# OR client# OR care OR healthcare OR " health care" OR service OR treatment ) N2 (experience* OR satisfaction OR assessment* OR evaluat* OR rating* OR opinion* OR judg* OR perception* OR perceive# OR perspective* OR "point-of-view")) OR (("patient reported" OR "self reported" OR patientreported OR selfreported ) W0 (satisfaction OR experience* OR perception# OR preference* )) OR PREM OR PREMs ) ) | 297,905 |
| S7 | (MH "Patient Satisfaction") | 63,650 |
| S8 | S6 OR S7 | 334,015 |
| S9 | (MH "Patient-Reported Outcomes") | 8,610 |
| S10 | TI ( (((consumer# OR patient# OR user# OR client# OR care OR healthcare OR "health care" OR service OR treatment ) N2 (("patient reported" OR "self reported" OR patientreported OR selfreported ) W0 (outcome* OR result# ))) OR PROM OR PROMs ) ) ) OR AB ( (((consumer# OR patient# OR user# OR client# OR care OR healthcare OR "health care" OR service OR treatment ) N2 (("patient reported" OR "self reported" OR patientreported OR selfreported ) W0 (outcome* OR result# ))) OR PROM OR PROMs ) ) ) | 19,201 |
| S11 | S9 OR S10 | 22,986 |
| S12 | S5 AND S8 AND S11 | 87 |
| S13 | EM 20231201- OR (RD 20231201- AND ZD "in process") OR PY 2023-2024 | 854,082 |
| S14 | S12 AND S13 | 22 |
