# Supplementary material for: Concurrent use and association of patient-reported experience and outcome measures in psychiatric and substance use disorder care: a scoping review
Source: Front Health Serv. 2025 Jun 30;5:1620809. doi: 10.3389/frhs.2025.1620809 (PMC12256552; doi:10.3389/frhs.2025.1620809)
Supplement: Supplementary file 3 [file Table3.docx]

**Appendix 3**

| **Supplementary table 1**. Studies excluded at full text screening | | | |
| --- | --- | --- | --- |
| Authors (year) | Country | Title | Reason for exclusion |
| Jansen L, Glowacz F,  Kinard A,  Bruffaerts R. (2022) | Belgium | How to Integrate Patient-Centered Measures in Routine Care: Lessons from Belgian Experiences | No full text available, only conference abstract |
| Killaspy H, Marston L,  Omar RZ, Green N,  Harrison I, Lean M et al. (2013) | England | Service quality and clinical outcomes: an example from mental health rehabilitation services in England | Not reporting on associations between PREMs and PROMs |
| Yamaguchi S, Ojio Y, Koike J, Matsunaga A, Ogawa M, Kikuchi A, et al. (2024) | Japan | Associations between readmission and patient-reported measures in acute psychiatric inpatients: a multicenter prospective longitudinal study | Not reporting on associations between PREMs and PROMs |
| Ruud T, Hasselberg N, Siqveland J, Holgersen KH. (2024) | Norway | Patient-reported outcome, clinician-reported outcome, and patient satisfaction with treatment by crisis resolution teams: a multicenter pre-post study of outcome and associated factors in Norway. | Not reporting on associations between PREMs and PROMs |

| **Supplementary table 2.** Characteristics of included studies | | | | | | | | |
| --- | --- | --- | --- | --- | --- | --- | --- | --- |
| Authors (year) | Country | Title | Objective | Context | Participants (Number, age, sex) | Questionnaires applied PREMS  (domains of experiences assessed) | Questionnaires applied PROMS | Results relevant for scoping review |
| Scanferla (2023) | France | How subjective well-being, patient-reported clinical improvement (PROMs) and experience of care (PREMs) relate in an acute psychiatric care setting? | To explore whether subjective well-being indicators (generic PROMs) are relevant for evaluating the quality of hospital care, distinct from measures of symptom improvement (disease-specific PROMs) and from PREMs | University psychiatric hospital, two departments (one department specialized in mood and eating disorders, one general psychiatric department providing care to patients suffering from diverse severe psychiatric disorders) | N=248 (final sample)  Mean age: 37.0 years  Female: 74.2%  30.6% suffered from eating disorders, 29.0% from psychotic disorders, 18.5% from mood disorders, 17.8% were hospitalized for suicidal crises and 4.0% suffered from alcohol-use disorder | Treatment satisfaction measured using a four-item rating scale adapted from the OECD-Proposed Set of Questions on Patient Experiences with Ambulatory Care  Dimensions explored: courtesy and respect; time spent with the clinician; clarity of the explanations and involvement in decisions about care and treatment | WHO-5  The OECD Assessment of Subjective Well-being Core Items (two items) module | Across mental disorders improvement in subjective well-being was only weakly correlated to experience of care.  The results support the case for using PROMs of subjective well-being in clinical practice as relevant indicators in their own right for patients undergoing psychiatric hospital treatment |
| Mendlovic (2022) | Israel | Exploring the relation between clinician ratings and patient-reported experience and outcomes | To investigate the associations between PROMs, PREMs and various symptoms measures reported by clinicians and psychiatric patients | Psychiatric hospital | N= 125  Men: 51.2%  Mean age: 44.4 years  34 % suffered from schizophrenia, 19% from depression, 3% from PTSD, and 44 % from personality disorders | Danish national mental health PROM programme (PRO-Psychiatry) (for experience with primary clinician)  Four items (focus on experiences of being respected, involved and receiving sufficient information) from the Consumer Assessment of Healthcare Providers and Systems programme, Agency for Healthcare Research and Quality (for experience with the treatment team) | Shortened version of the Manchester Short Assessment of Quality of Life (MANSA) | There was a high correlation between both PREMS measures and Quality of life (generic PROMS) |
| Liebmann (2022) | USA | Associations between patient experience and clinical outcomes in substance use disorder clinics: Findings from the veterans outcomes assessment survey | To provide information on Veterans Health Administration SUD specialty care at treatment initiation and approximately 3-months post-initiation | Veterans Health Administration outpatient clinics for substance abuse disorders | Participants in the Veterans Outcome Assessment (VOA) survey, one of three waves  N=2788  Male: 92%  Mean age: 50.7 year | The 6-item Communication Quality subscale of the ECHO  Measure of overall satisfaction adapted from the ECHO survey | Mental Health component score of the Short-Form-12 (MCS-12); index of overall emotional well-being | Problems with communication and overall satisfaction were both related to slightly lower scores of overall emotional well-being |
| Coelho (2022) | Portugal | Mental Health Patient-Reported Outcomes and Experiences Assessment in Portugal | To capture patient-reported outcomes and experiences on mental health care in Portugal using methods developed for international benchmarking purposes, such as the OECD Patient-reported Indicators Surveys. | Mental health care  Hospital outpatient setting/outpatient consultation or day hospital; 69.2%  Hospital inpatient setting: 6.3%  Community outpatient setting: 33.5% | N=397  Female: 62.2%  16–24 years: 5.6%  25–44 years: 31.2%  45–65 years: 51.1%  66 years or older: 12.1% | 4 items following OECD protocol:  Did your care providers treat you with courtesy and respect?  Did your care providers spend enough time with you?  Did your care providers explain things in a way that was easy to understand?  Did your care providers involve you as much as you wanted to be in decisions about your care and treatment? | WHO-5 Well-Being Index  In addition to the WHO-5, two items were included on life satisfaction and finding meaning in life, and four PREMS items on whether the treatment contributes to patient’s well-being and life satisfaction | Treatment satisfaction was associated with well-being |
