# Supplementary material for: Concurrent use and association of patient-reported experience and outcome measures in psychiatric and substance use disorder care: a scoping review
Source: Front Health Serv. 2025 Jun 30;5:1620809. doi: 10.3389/frhs.2025.1620809 (PMC12256552; doi:10.3389/frhs.2025.1620809)
Supplement: Supplementary file 4 [file Table4.docx]

| **Appendix 4.**  Data extraction form, modified from Peters et al, 2020* | |
| --- | --- |
| **Scoping Review Details** |  |
| Author |  |
| Publication year |  |
| Title |  |
| Objective |  |
| Country |  |
| Context |  |
| Participants (Number, age, sex) |  |
| **Details/Results** |  |
| Questionnaire(s) applied PREMs |  |
| Domains of experiences assessed |  |
| Questionnaire(s) applied PROMs |  |
| Results (relationship between PREMs og PROMs) |  |

* Peters MD, Godfrey C, McInerney P, Munn Z, Tricco AC, Khalil H. Scoping reviews. In: Aromatis E MZ, editor. JBI Manual for Evidence Synthesis: Joanna Briggs Institute; 2020. p. 1-24.
